# Characterization of total phenolic and flavonoid content in *Pseudoconyza viscosa* (Mill.) and its in-silico antioxidant evaluation

**DOI:** 10.3389/fpls.2025.1527515

**Published:** 2025-07-23

**Authors:** Ayesha Zahid, Abrar Hussain, Asim Mehmood, Muhammad Adnan Iqbal, Muhammad Akram, Javaid Iqbal, Mahmoud F. Seleiman, Nawab Ali, Adel M. Al-Saif

**Affiliations:** ^1^ Department of Chemistry, University of Agriculture Faisalabad, Faisalabad, Pakistan; ^2^ Department of Biosciences, COMSATS University Islamabad, Sahiwal, Pakistan; ^3^ Department of Environmental Sciences, COMSATS University Islamabad, Vehari, Pakistan; ^4^ Department of Plant Protection, College of Food and Agriculture Sciences, King Saud University, Riyadh, Saudi Arabia; ^5^ Department of Plant Production, College of Food and Agriculture Sciences, King Saud University, Riyadh, Saudi Arabia; ^6^ Department of Biosystems and Agricultural Engineering (BAE), College of Agriculture and Natural Resources, Michigan State University, East Lansing, MI, United States

**Keywords:** antioxidant potential, HPLC and UV-spectroscopy, molecular docking, total phenolic and total flavonoid content, *Pseudoconyza viscosa*

## Abstract

**Introduction:**

The *Pseudoconyza viscosa* (Mill.) is an important medicinal plant found in tropical and warm areas, and is known for its application in the food, cosmetic, and pharmaceutical industries. However, no information is available regarding its chemical composition, total phenolics and antioxidant potential. Thus, the present study aimed to investigate the total phenolic and flavonoid contents and their antioxidant potential through insilico studies.

**Methods:**

The ethanolic extracts were characterized by high-pressure liquid chromatography (HPLC) and UV-visible spectroscopy. Seventeen peaks were detected based on UV- spectroscopy. Furthermore, molecular docking of major phenolic compounds was carried out using Autodock Vina Software against human peroxiredoxin 5 (PDB ID: 1HD2) to study its antioxidant potential. Further, ADME predictions were made to determine physiochemical characteristics of the lead compound.

**Results:**

The total phenolic and flavonoid contents of *Pseudoconyza viscosa* (Mill.) ethanolic extracts were 311.74 and 208.2 mg/g respectively. Molecular docking results showed that dicaffeoylquinic acid (docking score -7.8) has significant binding potential against human peroxiredoxin 5 (PDB ID: 1HD2). ADME and drug likeness parameters have also shown that dicaffeoylquinic acid can be used as a potential antioxidant candidate compared to synthetic antioxidant drugs with side effects.

**Discussion:**

The results of this study underscore the therapeutic potential of *Pseudoconyza viscosa* (Mill.), warranting further investigation into its bioactive compounds for potential applications in pharmaceuticals and nutraceuticals. Future research should focus on exploring the mechanisms and efficacy of these compounds in clinical settings, paving the way for the development of novel therapeutic agents derived from this medicinal plant.

## 1 Introduction

Herbal medicinal plants and their essential oils are commonly used to treat various ailments in developing countries (Jamshidi-Kia et al., 2017). The content of essential oils varies among different plant parts; however, more aerial and flower parts are reported to have a higher content because of more oil-producing glands. These essential oils are used in the food industry as flavoring agents, therapeutic agents to treat upper respiratory tract infections, and fragrances in the cosmetic industry. Due to the presence of antioxidative substances in essential oils, oxidative damage and inflammatory diseases of the cell can be prevented. Phenolic compounds present in essential oils contain large quantity of flavonoids which are known to have biological and antioxidant properties (Boukhatem and Setzer, 2020; Godlewska-Żyłkiewicz et al., 2020). Flavones, catechins and flavenols are among the most common flavonoids found in plants and exhibit anti-cancer, anti-inflammatory and antioxidant activities (Panche et al., 2016; Sen et al., 2013).

In the present age, entry of toxic compounds through the consumption of food and drinking water results in the production of free radicals which ultimately cause different diseases in humans. Free radicals react with fatty acids, lipids, DNA, proteins and damage the cell membranes. The concentrations and specific profiles of these bioactive compounds can vary significantly across plant species, plant parts, and environmental growth conditions (Lobo et al., 2010). Furthermore, the biological activities of plant-derived phenolic and flavonoid compounds are closely linked to their molecular interactions with various protein targets involved in disease pathogenesis (Arya and Coumar, 2021). Moreover, advancements in metabolomics have significantly improved the understanding, and more integrated analyses of metabolic processes in plant compounds such as phenolics and flavonoids (Yang et al., 2024).

*Pseudoconyza viscosa* (Mill.) is an herbal plant found in hot areas and is famous for its medicinal importance. *Pseudoconyza viscosa* (Mill.), commonly referred to as zahika, belongs to the Asteraceae family. It is cultivated in tropical regions of South America and Antarctica. The plant height is from 1.0 to 2.0 meters and exhibits a range of colors at maturity, including purple and whitish-blue (Jung et al., 2009). Despite its cultivation, there is limited information available on its anticancer, antioxidant, and antimicrobial properties (Malami et al., 2016).

Molecular docking is a computational method used to predict the binding affinity and interaction mode between small molecules and macromolecular targets, thereby offering valuable insights into the potential therapeutic mechanisms of natural products (Gupta et al., 2018; Liu et al., 2018). It is important to characterize the phenolic and flavonoid contents of medicinal plants such as *Pseudoconyza viscosa* (Mill.) to better understand their potential therapeutic applications.

The compounds derived from this plant are mainly oxygenated compounds, in which alkaloids, monoterpenes, and sesquiterpenes cover the main portion (Wu et al., 2006). The essential oils can be obtained from different parts like root, stalk and from its leaves. Based on the nutraceutical and therapeutic potential of essential oil from *Pseudoconyza viscosa* (Mill.), the present study was planned to study its total phenolic and flavonoid content. Docking analysis justifies the observed antioxidant activities by simulating interactions between bioactive compounds and relevant molecular targets. This approach not only validates experimental results but also supports the plant’s potential as a source for pharmaceutical development. Molecular docking analysis was conducted using human peroxiredoxin 5 (PDB ID: 1HD2) to evaluate its antioxidant potential. Additionally, ADME predictions were performed to ascertain the physicochemical characteristics of the lead compound. Considering the nutraceutical and pharmacological properties of *P. viscosa* (Mill.), it is imperative to conduct a critical evaluation of previous research to guide the design of future studies. Thus, the present study aims to address this gap by examining the chemical composition, phenolic profile and antioxidant potential of the methanolic extract obtained from *P. viscosa* (Mill.) which is extensively utilized in traditional medicine, and to discuss the potential use in pharmaceutical industry.

## 2 Materials and methods

### 2.1 Plant material and extraction

**Figure 1:**
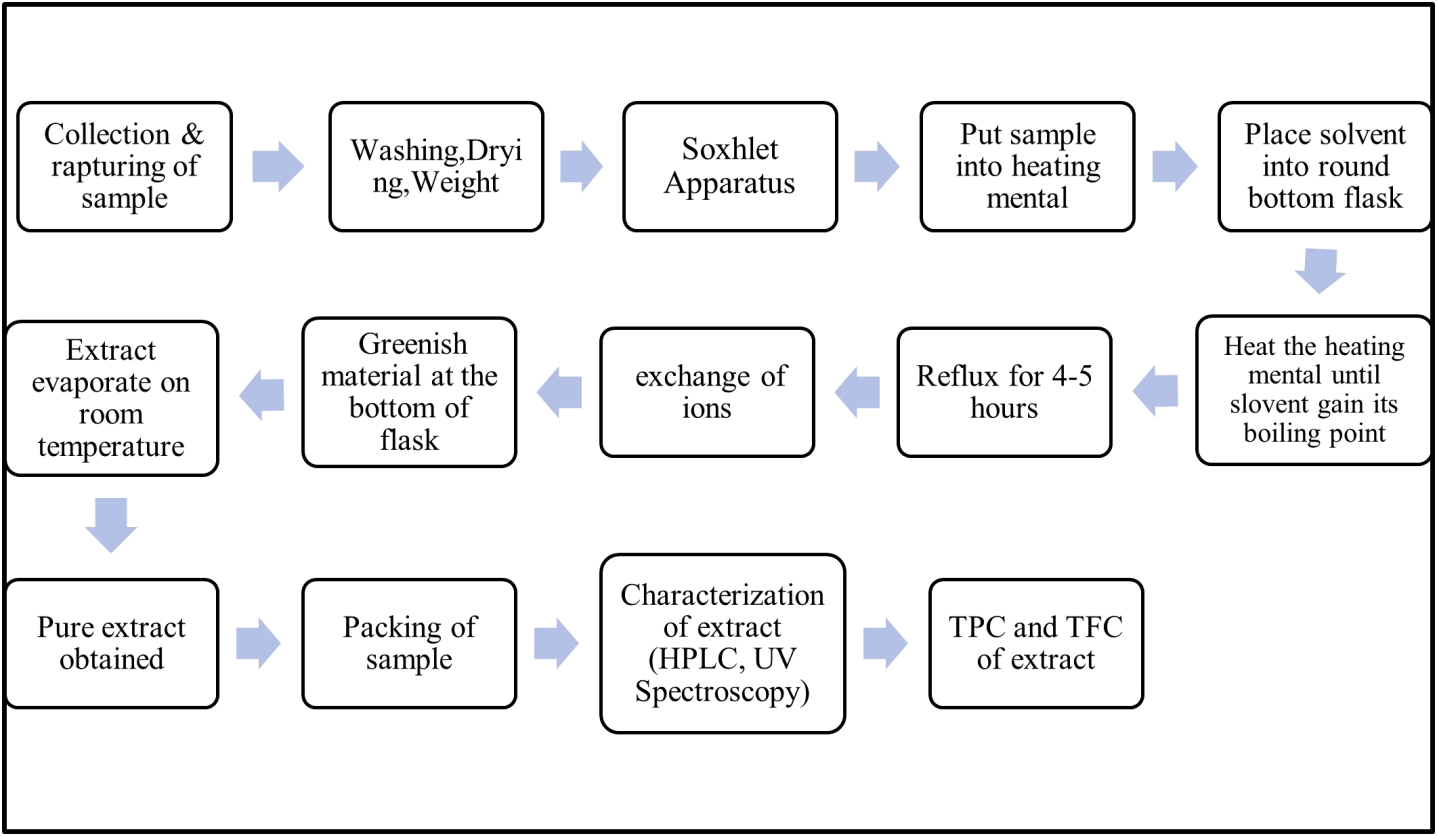
Schematic diagram for the extraction and analysis of total phenols and flavonoids.

The plant was collected from District Dera Ghazi Khan Punjab Pakistan (30° 2’ N and 70° 38’ E). The solvents used for the extraction were ethanol, ether, and chloroform, whereas sodium carbonate, aluminum chloride, folin reagent, and sodium nitrate were used for TPC and TFC (Handa, 2005). About 50 g of plant sample was weighed and grinded in pester mortar with a small amount of ethanol. The paste of plant sample was packed into Soxhlet apparatus for extraction. The greenish extract was the product of solvent extraction, which further proceeds for decolorization by different concentrations of activated charcoal. Steps involved in the extraction and analysis are presented in **Figure 1**.

### 2.2 Total phenolic contents

The TPC of the samples was estimated using the folin-Ciocalteu reagent method described by (Ozturk et al., 2022). Briefly, the total phenolic content of the plant extract was determined on the basis of a dilution method about to 0.2 N in the presence of folin-Ciocalteu reagent, and kept for 2 h in the culture tube. This material was further oxidized using sodium carbonate at approximately 75/g per liter. The absorbance of the sample was measured using a spectrophotometer at 750–760 nm using gallic acid as the standard reagent (Ozturk et al., 2022).

### 2.3 Total flavonoids contents

TFC contents were measured according to the method described by Sen et al (Sen et al., 2013). A volume of 250 ml plant extract added in culture tube containing distilled water. Then, 76 µL sodium nitrate was added to the culture tube. Further AlCl_3_ was added in the culture tube for 6–7 minutes. After this, the plate was placed in the spectrophotometer and reading was recorded at 500 nm. Three readings were taken. Quercetin was used as a standard reagent in the calibration curve (Dewanto et al., 2002).

### 2.4 UV-VIS analysis

The extracts were subjected to UV-VIS scanning and diluted 30 times with the appropriate extraction solvent (ethanol or methanol) at room temperature before analysis. Absorbance spectra were acquired between 200 and 400 nm using a UV-VIS spectrophotometer.

### 2.5 HPLC analysis

The extracts of *Pseudoconyza viscosa* (Mill.) were analyzed using an HPLC system (YL 9100, Korea), which included an autosampler (YL 9150) with a 100 µL fixed loop and a YL 9120 UV-visible detector. Separation was carried out on an SGE Protecol PC18GP120 column (250 mm × 4.6 mm, 5 µm) at ambient temperature. The mobile phase consisted of methanol and water in a 70:30 (v/v) ratio, and the separations were conducted in iso-cratic mode with an elution flow rate of 1 mL/min. Standards were used to validate the peaks.

### 2.6 Molecular docking investigations

**Figure 2:**
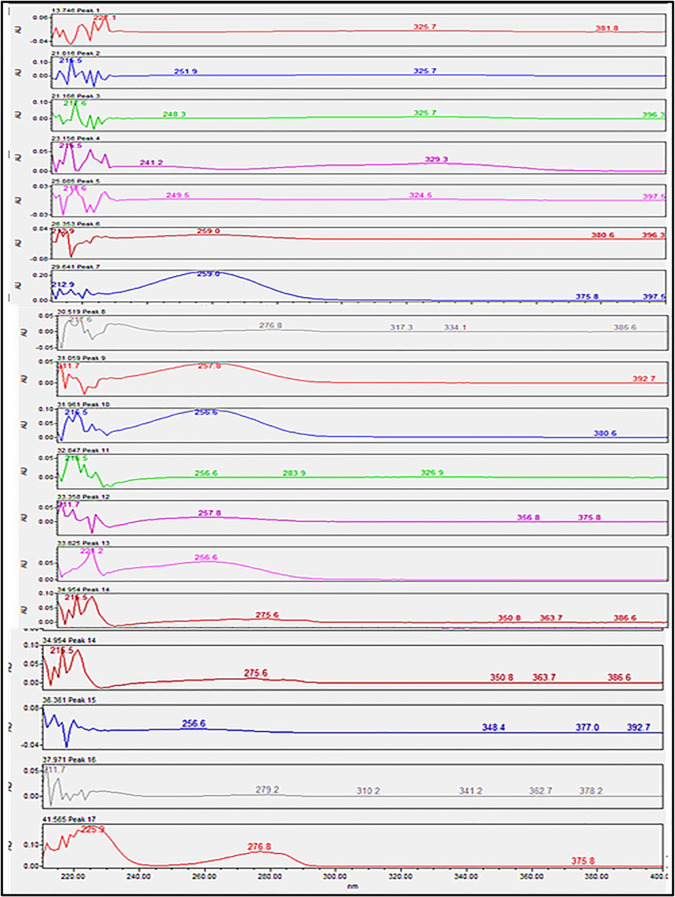
UV spectrum of the identified compounds of ethanolic extract of *Pseudoconyza viscosa* (Mill.).

Molecular docking studies explained the behavior of ligands isolated from *Pseudoconyza viscosa (*Mill*.)* against functional residues involved in the antioxidant activity of the human peroxiredoxin 5 protein using a dataset of 11 phytochemicals (**Table 1**). The structure of target protein was retrieved from the Protein Data Bank (PDB ID:1HD2) having high resolution 3.78 Å, ligand bound in the active site and no mutation as shown in **Figure 2**. AutoDock Vina was used to perform molecular docking analysis. This standalone offline tool works in two modes: (i) MGL tools GUI based system, used for receptor (protein) and ligand *Pseudoconyza viscosa (*Mill*.)* compounds preparation. (ii) Command based mode used to execute the ligand -receptor docking (Gupta et al., 2018).

**Table 1:** Compounds identified by UV spectroscopy, confirmed and downloaded from PubChem.

Sr. No	Substance	PubChem ID	Lambda Max (nm)	Molar mass (g/mol)
Peak 1.	Phenanthrene	995	227.1	178.23
Peak 2	Dicaffeyl derivative	12358846	216.5	388
Peak 3.	O-coumaric acid	637540	217.6	164.16
Peak 4.	Coumyl quinic acid	6508	216.5	192.17
Peak 5.	Feroyl quinic acid	9799386	217.6	368.3
Peak 6.	Cichoric acid	5281764	212.9	474.371
Peak 7.	Vanillic acid	8468	212.9	168.14
Peak 8	Quercetin-O-hexoside	44259229	217.6	464.09
Peak 9	4-Methyl-3-methoxy-9-ahydroxyligballinol-O-glucoside	27882	211.7	137.18
Peak 10	Hydroxycinnamic acid derivative	637541	216.5	137.18
Peak 11	Hydroxycinnamic acid	637541	216.5	137.18
Peak 12	Quercetin-3-O-rutinoside	5293655	211.7	610.51
Peak 13	Kaempferol-3-O-rutinoside I	5318767	221.2	594.52
Peak 14	Dicaffeoylquinic acid IV	12358846	216.5	516.4
Peak 15	Dicaffeoylquinic acid	12358846	216.5	516.4
Peak 16	Kaempferol-3-O-rutinoside II	5318767	211.7	594.52
Peak 17	Dicaffeoylquinic acid V	12358846	225.9	516.12

#### 2.6.1 Ligand preparations

The pdb file of ligand was imported to MGL tools. Torsion roots were detected, and torsion angles were adjusted. Gasteiger charge was added and Kollman charge was computed. The prepared ligand was saved in pdbqt format.

#### 2.6.2 Receptor protein preparation

The pdb protein file was imported to MGL tools. Protein was refined by removing, non-standard amino acids, het atoms & water molecules, and adding polar hydrogen. The prepared molecule was saved in pdbqt format. Three-dimensional grid for the ligand attachment pocket was adjusted, and the 3D grid dimensions were saved as a grid.txt file. A config.txt file, containing information about receptor, ligand names and grid dimensions, was prepared.

#### 2.6.3 Docking analysis

The ligand-protein docking was performed in command line mode of AutoDock Vina. The command containing information about directories and config file was run and resultant log files and out,pdbqt files were retrieved.

#### 2.6.4 Creation of docked complex and interaction visualization

The out.pdbqt and protein.pdb files were opened in PYMol and merged to create complex.pdb file for ligand-protein interaction visualization. The complex file was opened in LigPlot for 2D interactions visualization while for 3D interaction visualization it was opened in BIOVA Discovery studio.

#### 2.6.5 Lead identification

The log file contains information about RMSD and binding affinities for 10 different possess for each compound. The molecules showing the lowest binding affinities in complex and highest number of hydrogen bonds were selected as lead compound for respective protein.

#### 2.6.6 Pharmacophore modelling

The top chemical compounds with the lowest binding affinities for each protein were selected to generate the pharmacophore. The pharmacophores for merged and shared features of the selected compounds were built using LigandScout v. 4.4.9.

## 3 Results and discussion

### 3.1 UV-VIS analysis

The results of the UV-VIS Analysis showed the presence of several aromatic compounds. The absorption of electromagnetic radiation at 200–400 nm indicates the presence of heteroatoms. Compounds containing σ bonds, π bonds, a lone pair of electrons, chromophores, and aromatic rings were identified in UV UV-visible spectra. The spectra of the resultant compounds are shown in **Figure 2**.

The extract revealed the presence of various phenolic and flavonoid compounds including phenanthrenes (peak 1), dicaffeyl derivatives (peak 2), hydroxycinnamic acids (peaks 3-7), flavonoid glycosides (peaks 8-12), and dicaffeoylquinic acids (peaks 13-15). These findings are in consistent with earlier phytochemical research on other medicinal plants, which have reported it as a rich source of polyphenolic secondary metabolites (Lobo et al., 2010; Sen et al., 2013). Additionally, these compounds have demonstrated potent antioxidant and neuroprotective effects (Al-Khayri et al., 2022).

The identified flavonoid glycosides, such as quercetin, kaempferol, and their rutinoside derivatives, are well-known for their potent antioxidant, anti-inflammatory, and anti-cancer properties (Al-Khayri et al., 2022). Similarly, the dicaffeoylquinic acids detected (peaks 13, 15) demonstrate a broad spectrum of pharmacological activities, including anti-inflammatory, neuroprotective, and anti-diabetic effects (Boukhatem and Setzer, 2020; Marcos-Gómez et al., 2024). The presence of these derivatives in the P. *Pseudoconyza viscosa (*Mill*.)* extract suggests that they may contribute significantly to the observed biological activities of this medicinal plant. (**Table 1**).

### 3.2 HPLC analysis

**Figure 3:**
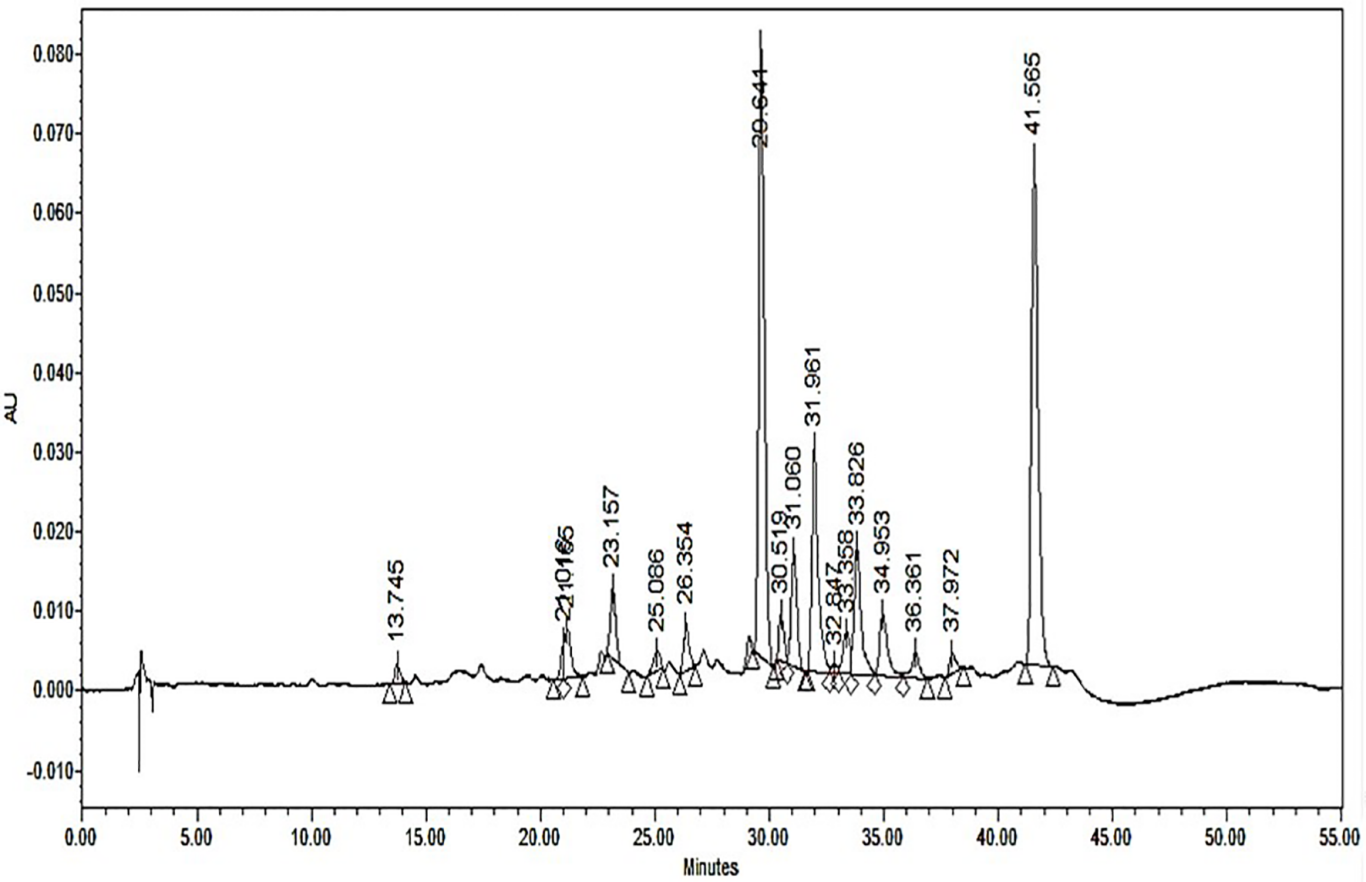
HPLC chromatogram of *Pseudoconyza viscosa* (Mill.) extract showing compounds 1.Malonyl-1,4-O-dicaffeoylquinic acid (RT 23.157 min), 2.Quercetin-O-coumaroylhexoside (RT 26.354 min), 3.Quercetin-O-dihexoside (RT 29.641 min), 4.Kaempferol-O-caffeoylhexoside (RT 31.060 min), 5.5-Hydroxyl-6,8-dimethoxy-7-hexoside flavone (RT 31.961 min), 6.5-Hydroxy-7,8,6’-trimehoxy-2’-hexoside(acetyl) flavone (RT 41.565 min).

The HPLC analysis of the ethanolic extract of *Pseudoconyza viscosa* (Mill.) revealed the presence of several phenolic and flavonoid compounds (**Figure 3**). The extraction of active components consists of different steps which can affect both the quality and quantity of the extracted components. The diversity of peaks suggests a complex chemical profile, which may explain the broad-spectrum antioxidants, antimicrobial, and enzyme inhibitory activities previously reported for *Pseudoconyza viscosa* (Mill.). Compounds eluting at earlier retention times are likely hydrophilic, such as phenolic acids, whereas those eluting later may be hydrophobic, including flavonoids or other secondary metabolites. In our analysis, Quercetin-O-dihexoside was the most abundant compound, with a concentration of 48.7 mg/L. It was closely followed by 5-Hydroxy-7,8,6’-trimethoxy-2’-hexoside(acetyl) flavone at 35.5 mg/L. The other compounds we measured included: Malonyl-1,4-O-dicaffeoylquinic acid (18.3 mg/L), Kaempferol-O-caffeoylhexoside (21.7 mg/L), and 5-Hydroxy-6,8-dimethoxy-7-hexoside flavone (23.5 mg/L) (**Table 2**).

**Table 2:** PubChem ID, chemical structure, docking score and hydrogen bond interactions of *Pseudoconyza viscosa* (Mill.) chemical compounds.

PubChem ID	Chemical structure	Docking score	Hydrogen bond interaction		
			Interaction	Distance	No of bonds
12358846	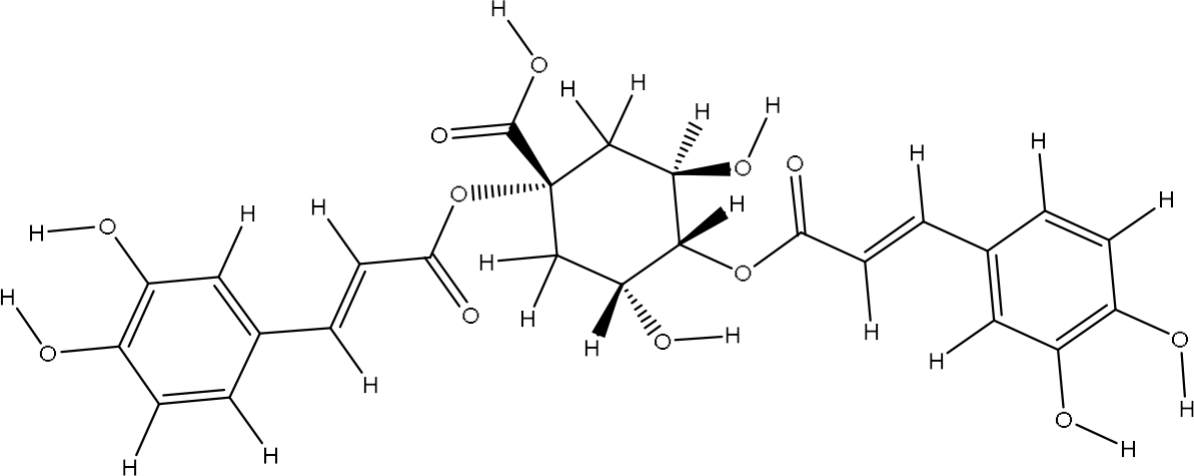	-7.8	Val70:N-O12 Val70:O-O12 Gly92:N-O8 Gly92:N-O9 Val94:O-O8 Ala90:O-O8 Leu96:N-O2	2.96 2.79 3.07 3.33 2.78 3.01 3.13	7
44259229	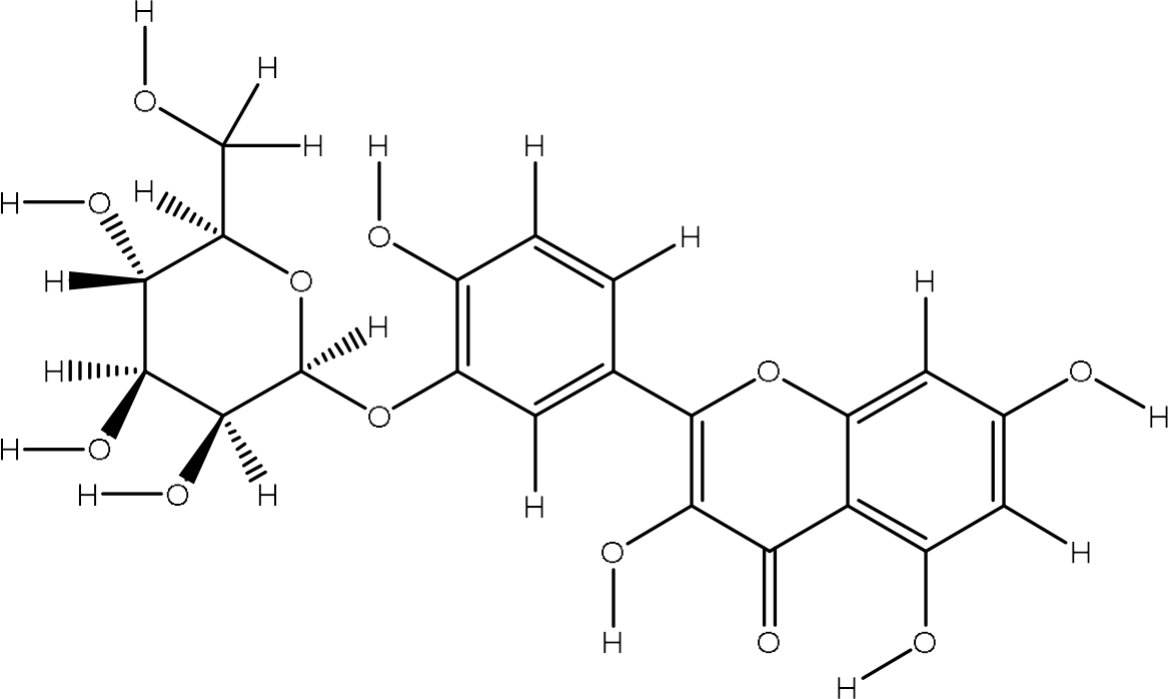	-7.5	Val70:N-O5 Gly82:O-O10 Leu96:O-O11	2.98 3.22 3.07	3
5318767	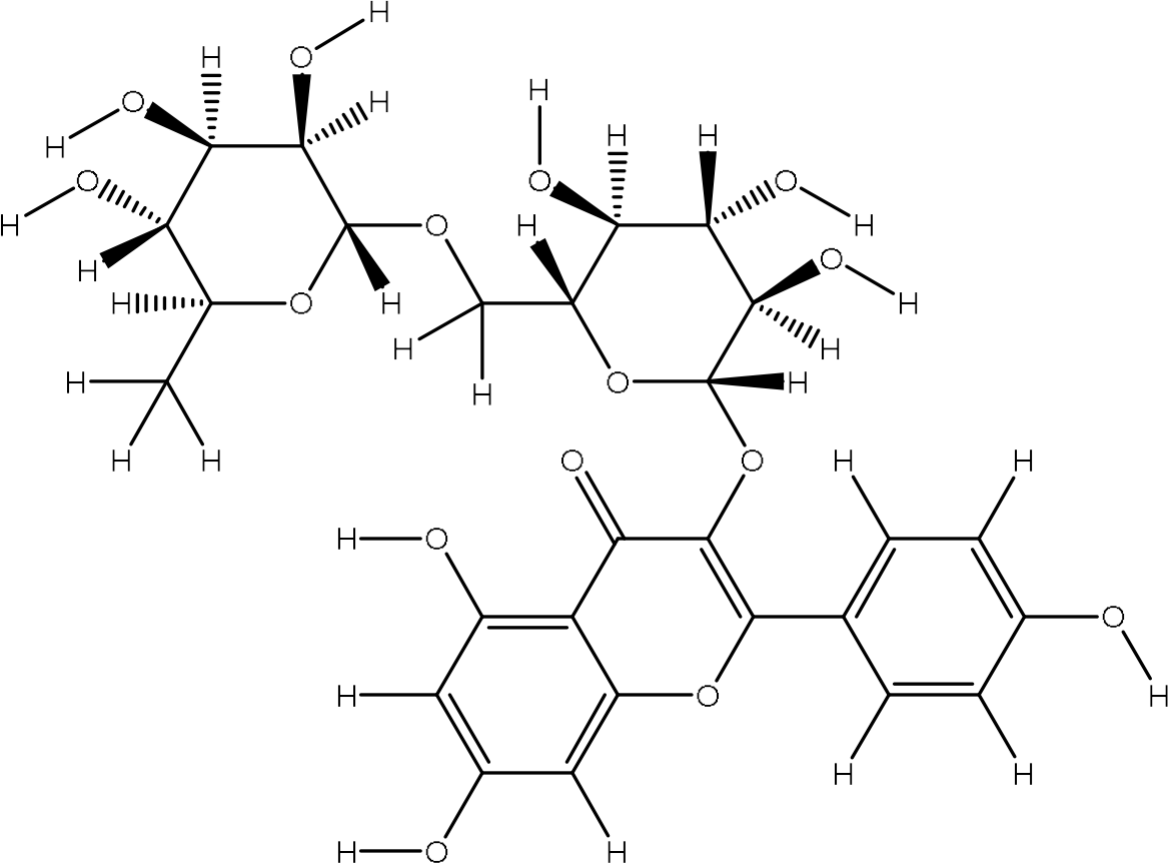	-7.4	Arg86:NH2-O8 Arg86:NH2-O9 Glu16:O-O15 Asn21:ND2-O15	3.18 2.95 2.97 2.94	4
5293655	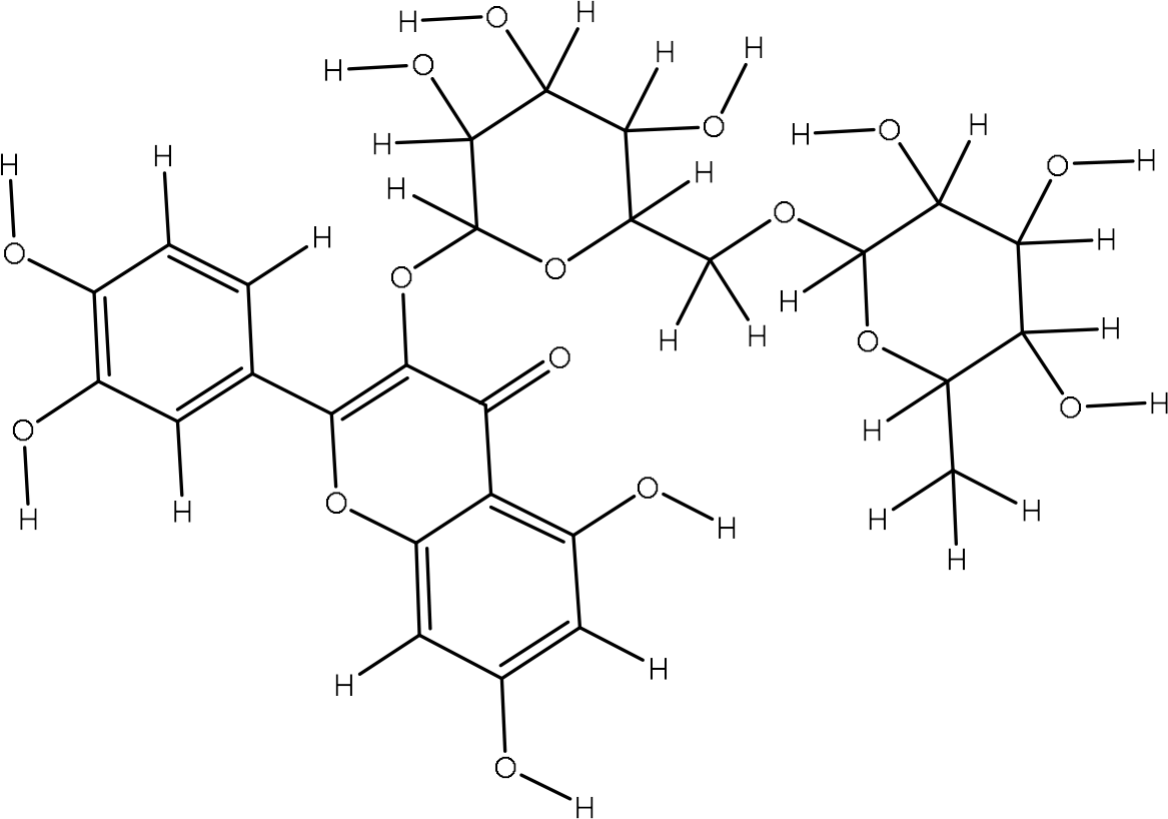	-7.4	Gly17:NH2-O5 Gly17:NH2-O6 Gly82:O-O12 Arg95:NH2-O7 Lys22:O-O8	2.87 3.07 3.00 3.24 2.92	5
9799386	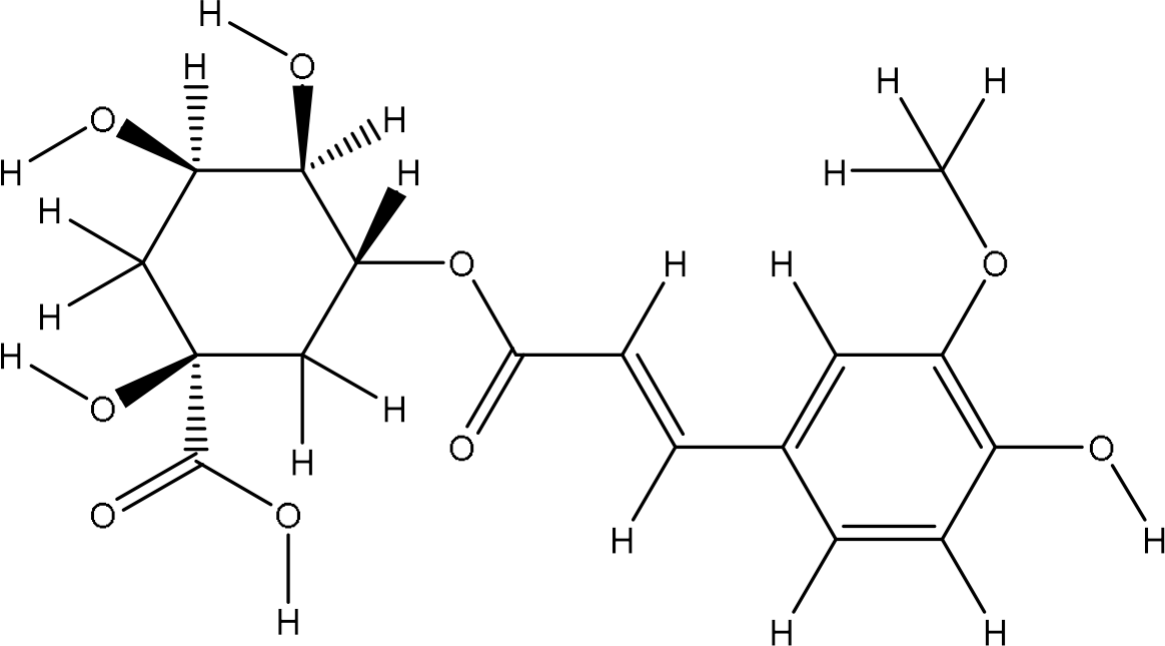	-6.7	Ala90:N-07 Glu91:N-O7 Glu16:OE2-O5 Leu96:N-O5 Leu96:O-O5	3.28 3.01 3.17 3.12 3.32	5
5281764	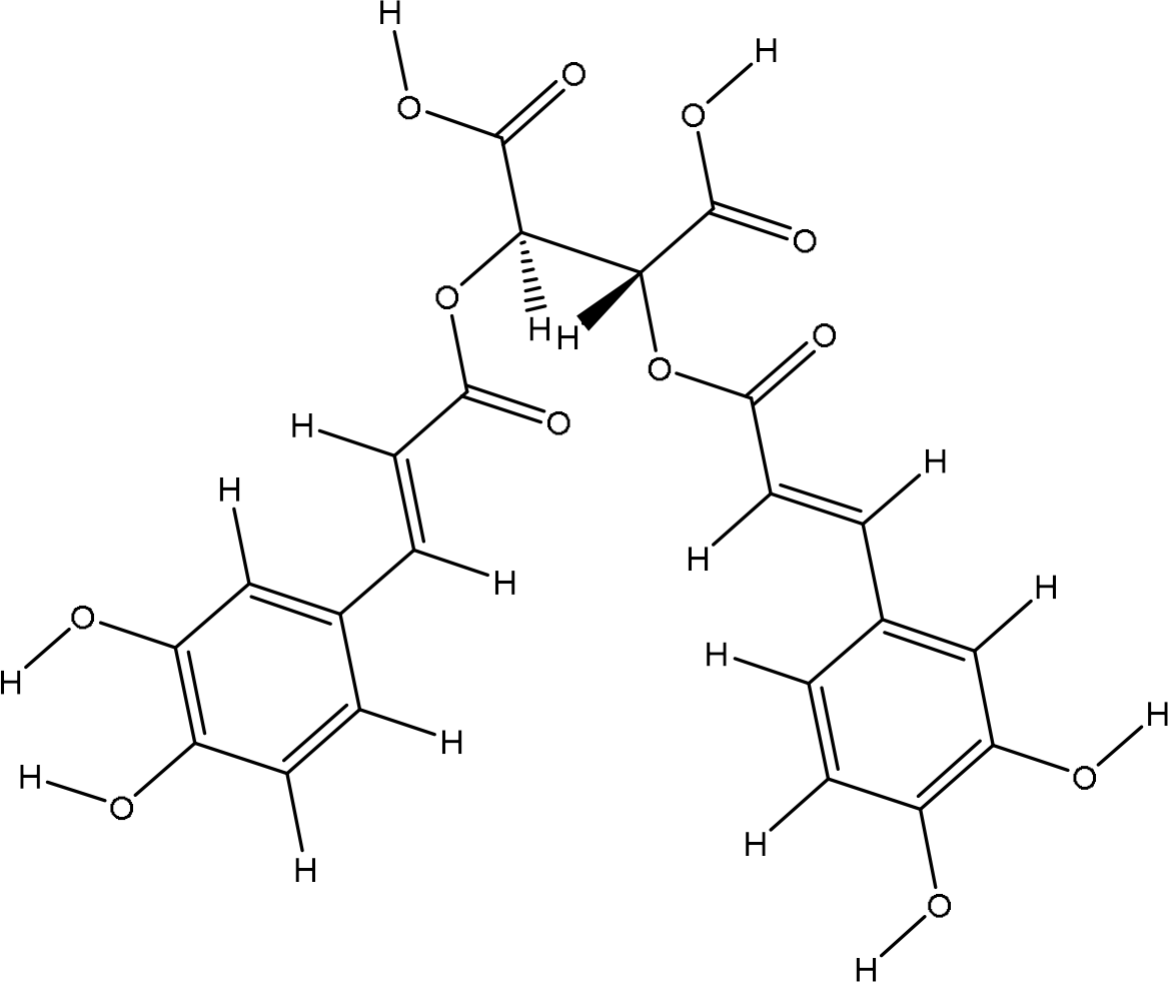	-6.1	Glu16:OE2-O6 Glu16:OE2-O11 Leu96:N-O11 Leu96:N-O12 Val94:O-O11	2.70 3.20 3.16 3.24 3.14	5
995	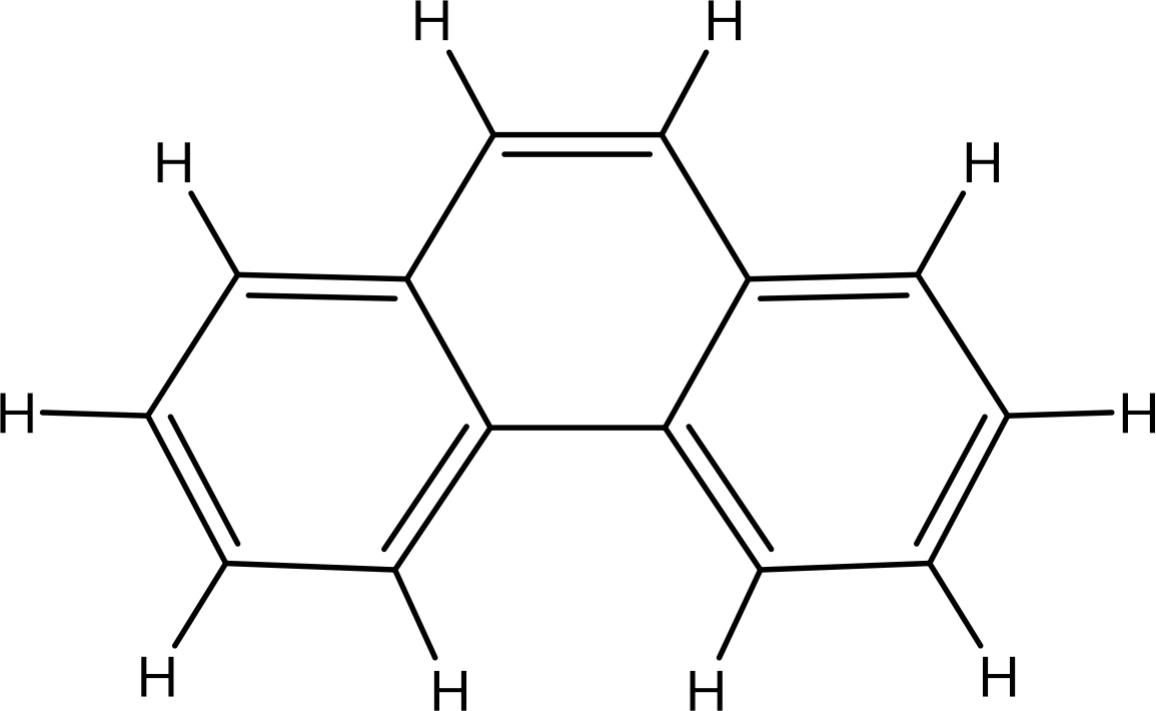	-5.9	No		0
637540	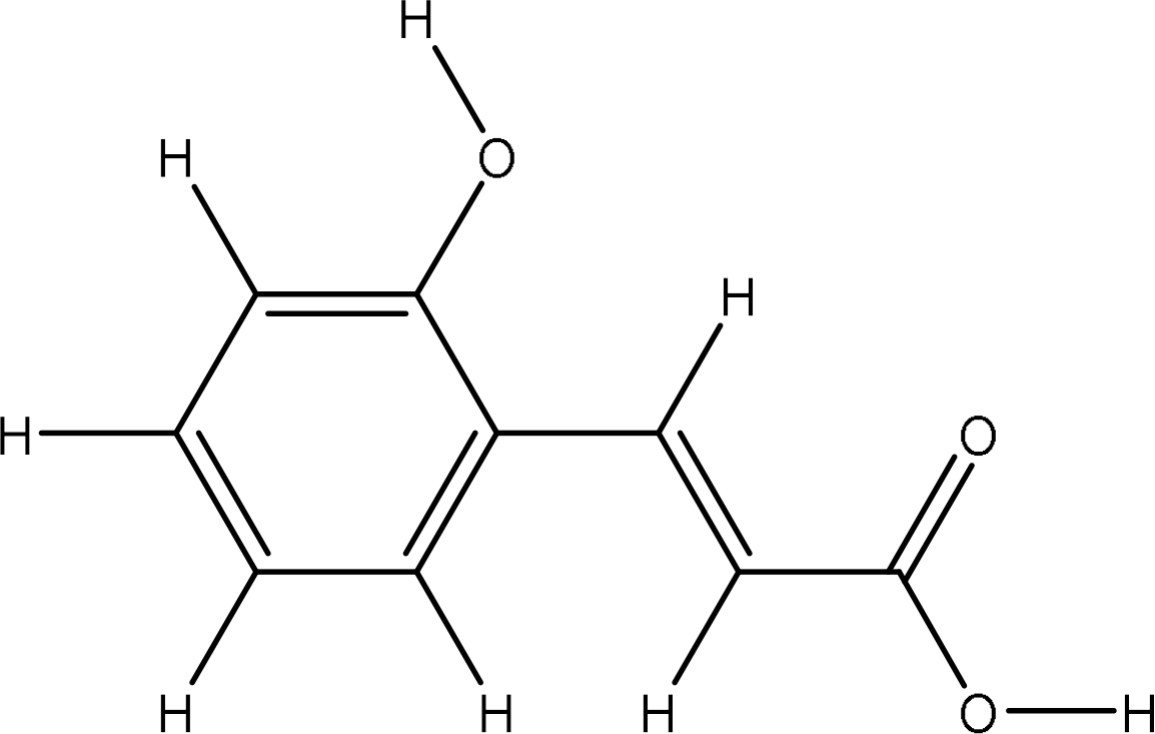	-5.8	Gly82:O-O1 Gly85:O-O3 Ala90:N-O3 Glu91:N-O3	2.71 3.09 2.91 3.01	4
8468	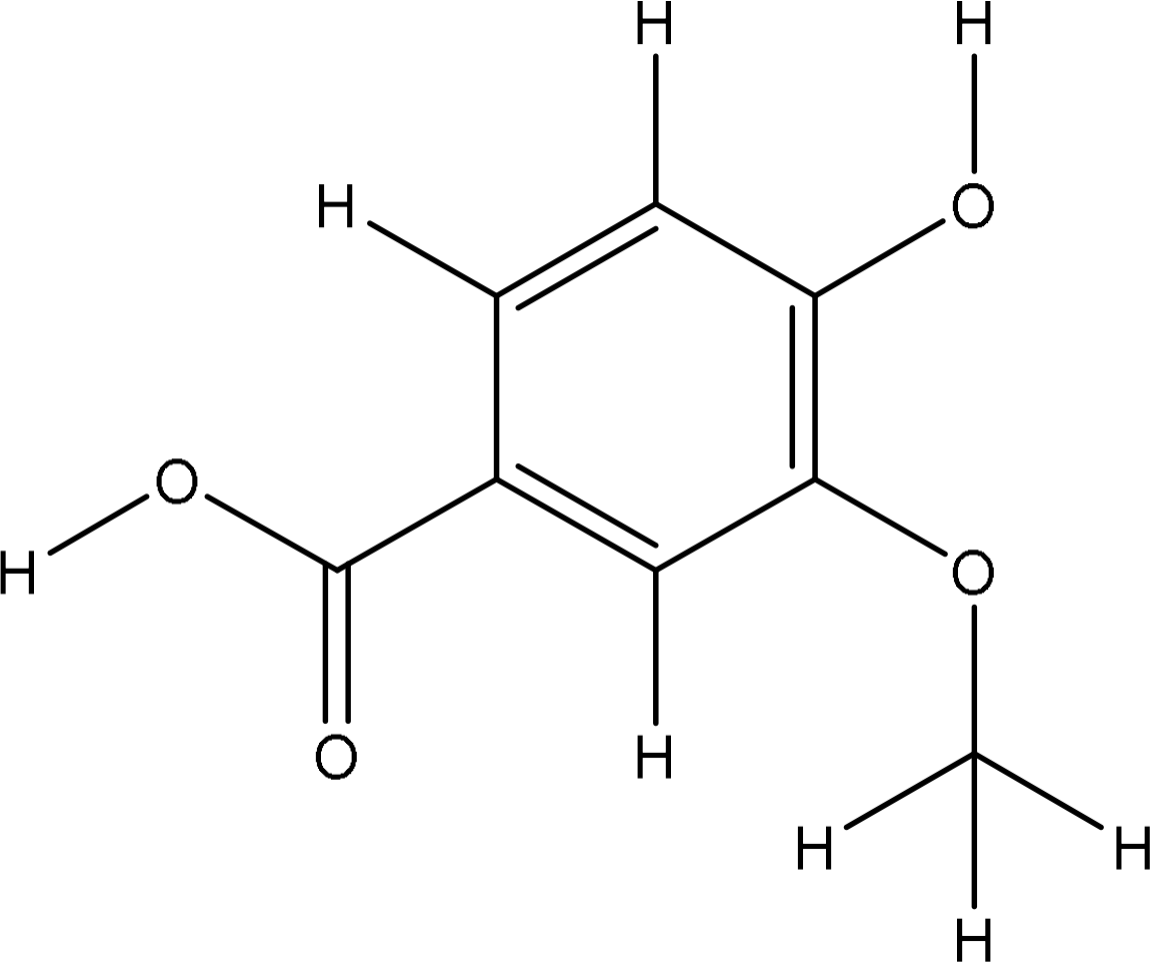	-5.8	Glu16:OE2-O4 Glu91:N-O2 Ala90:N-O2 Leu96:O-O4 Leu96:N-O4	3.11 2.94 3.13 3.12 2.86	5
6508	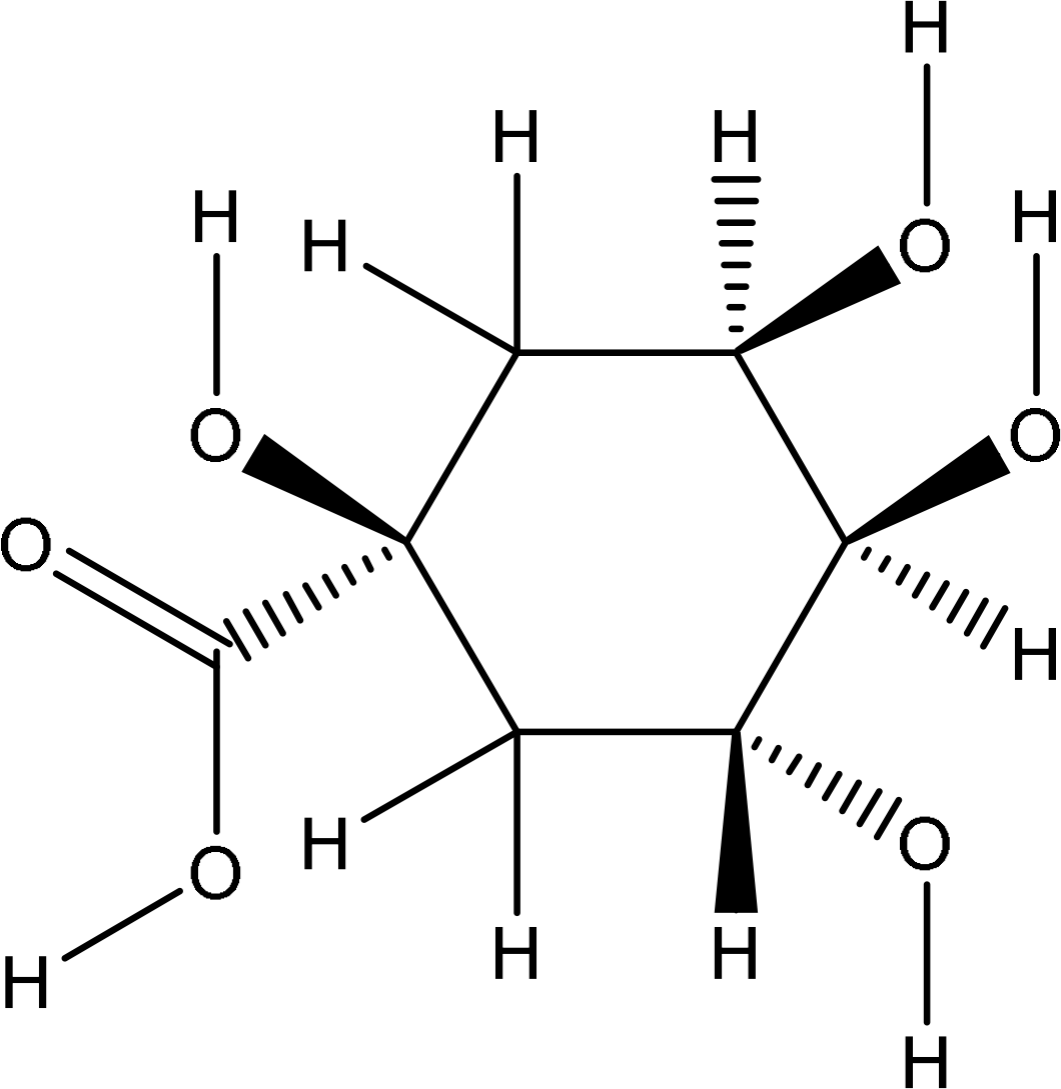	-5.7	Glu16:OE2-O4 Glu16:OE2-O6 Glu91:N-O2 Leu96:O-O6	3.14 3.19 3.03 3.18	4

Quercetin is well known for its antioxidant properties, primarily due to its ability to scavenge free radicals and mitigate oxidative stress (Qi et al., 2022; Deepika and Maurya, 2022). This observation is consistent with earlier studies suggesting that quercetin-rich compounds can offer various health benefits, including anti-inflammatory and anticancer effects (Shamsudin et al., 2022; Saeed et al., 2012).

Malonyl-1,4-O-dicaffeoylquinic acid and Kaempferol-O-caffeoylhexoside further enhance the antioxidant profile of *Pseudoconyza viscosa* (Mill.). Malonyl-1,4-O-dicaffeoylquinic acid, a derivative of chlorogenic acid, is linked to various pharmacological properties, including potent antioxidant activity and potential protective effects against neurodegenerative diseases (Szwajgier et al., 2017; Jang et al., 2022). Kaempferol and its derivatives have been found to have impact on inflammation and promoting cardiovascular health (Kamisah et al., 2023). 5-Hydroxyl-6,8-dimethoxy-7-hexoside flavone is not much studies however flavonoids with similar structures have been noted for their antimicrobial and antioxidant properties (Al-Khayri et al., 2022).

### 3.3 Total phenolic and flavonoids content

**Figure 4:**
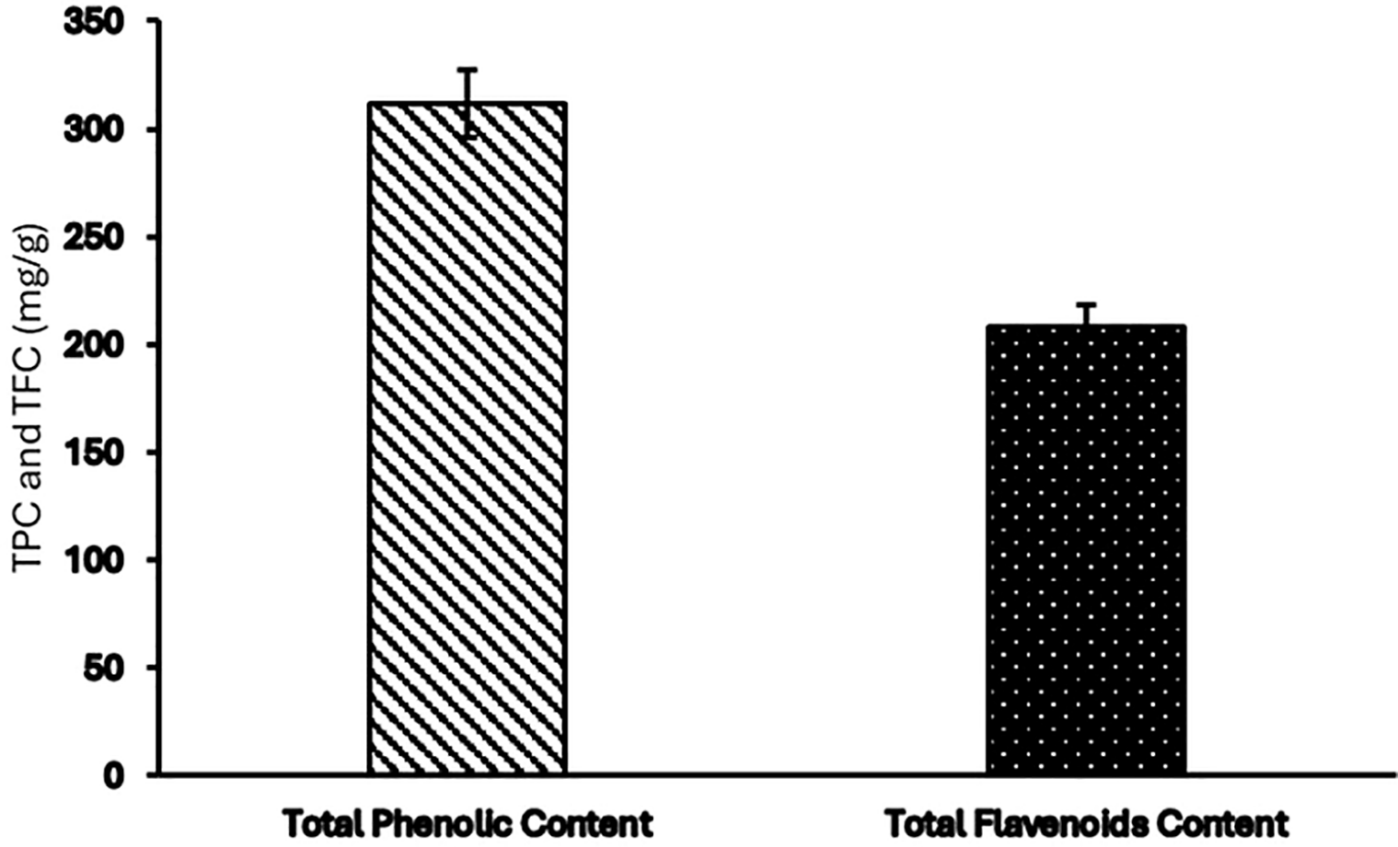
TPC and TFC contents of *Pseudoconyza viscosa* (Mill.) extract.

The ethanolic extract of *Pseudoconyza viscosa* (Mill.) demonstrates exceptional phytochemical richness, with quantitative analysis revealing remarkably high total phenolic content (TPC) of 311.74 mg GAE/g and total flavonoid content (TFC) of 208.2 mg QE/g (**Figure 4**). These values represent a significant phytochemical profile, substantially exceeding previously reported values for related species such as *Inula viscosa*, which showed TPC of 236.78 ± 7.63 mg GAE/g and TFC of 94.36 ± 1.86 mg QE/g in methanolic extract. The considerable difference-approximately 32% higher phenolic content and 121% higher flavonoid content-positions *P. viscosa* as an exceptionally potent source of these bioactive compounds among medicinal plants, suggesting enhanced therapeutic potential.

The high total phenolic and flavonoid contents observed in the *Pseudoconyza viscosa* (Mill.) (**Figure 4**).

Several studies have reported the presence of diverse phenolic and flavonoid compounds in *Pseudo Conyza* and related species. For example, a study on *Pseudoconyza anastomosans*, a closely related plant, found significant levels of phenolic acids, flavonoids, and other polyphenols (Karak, 2019; Mrid et al., 2022). Similarly, research on Conyza species, which are closely related to *Pseudoconyza*, has shown the abundance of phenolic and flavonoid constituents (Jung et al., 2009). The TPC and TFC values are notably higher compared to a previous study, which reported TPC at 236.78 ± 7.63 mg GAE/g and TFC at 94.36 ± 1.86 mg QE/g in the methanolic extract of *Inula viscosa* (Eruygur et al., 2024). The significant levels of phenolics and flavonoids in *P. viscosa* indicates its potential for various applications in the nutraceutical industry. Phenolic compounds are not only for its antioxidant properties but also for their antimicrobial and anti-inflammatory properties. Flavonoids also provide additional health benefits, such as enzyme inhibition and modulation of cell signaling pathways. These bioactive compounds in *P. viscosa* position it as a promising candidate for developing natural antioxidant supplements or therapeutic agents aimed at managing disorders related to oxidative stress.

### 3.4 Molecular docking investigations

**Figure 5:**
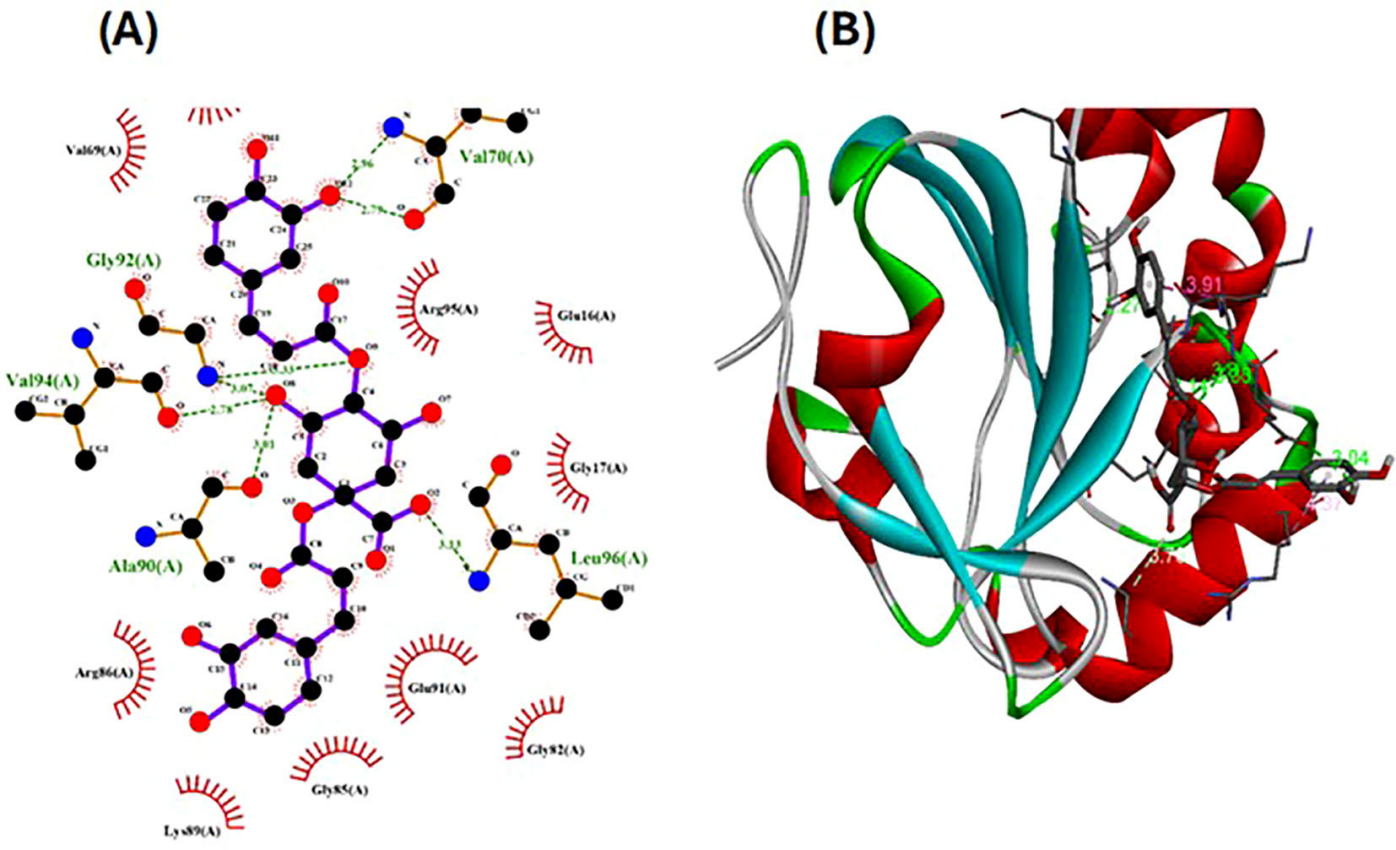
The ligand-receptor complexes (2D and 3D interactions) of *Pseudoconyza viscosa* (Mill.) chemical compounds. **(A)** Schematic representation of molecular interactions between ligand and the active site. **(B)** Ribbon diagram of protein structure highlighting secondary structural elements.

The pharmacophore model provides a valuable framework for investigating the structure-activity relationships and potential mechanisms of action of the phytochemicals found in the *Pseudoconyza viscosa* (Mill.) extract. This exploration may aid in developing therapeutic applications for this medicinal plant (Muhammed and Akı-Yalcın, 2021; Gao et al., 2010). Eleven compounds from *Pseudoconyza viscosa (Mill.)* were docked with human peroxiredoxin 5 protein and results of the top-scored 10 compounds were shown with their binding energies ranging from -7.8 to -5.7 kcal/mol and binding interaction within the range of 4.0 Å. Human peroxiredoxin 5 (PRDX5) is known to have various pathological functions associated with oxidative stress, including neurodegenerative diseases, cancer, and inflammatory disorders. The inhibitors of Human peroxiredoxin 5 were analyzed using LigPlot to determine the amino acids residues involved in protein-ligand interactions. Dicaffeoylquinic acid IV shows least binding affinity and maximum binding interactions as shown in **Figures 5a, b**. The pharmacophore model provides a valuable foundation for further optimization and structural modifications of the bioactive compounds isolated from *Pseudoconyza viscosa (Mill.)*. The insights gained can guide the design of novel derivatives with improved pharmacological properties, such as enhanced potency, selectivity, and bioavailability (Muhammed and Akı-Yalcın, 2021; Kumar and Bora, 2014). The structural features of 3,4-dicaffeoylquinic acid that enable its strong binding affinity include its dual caffeoyl substituents ester-linked at positions 3 and 4 of the quinic acid backbone, which introduce polarity and aromatic π-systems for hydrogen bonding and π-π stacking interactions. The catechol motifs in the caffeoyl groups enhance redox activity, stabilizing interactions in oxidative environments, while the quinic acid ring’s hydroxyl groups facilitate extensive hydrogen bonding with biological targets like enzymes or plasma proteins. The ester linkages contribute to structural rigidity and planarity, optimizing spatial alignment for interactions, and the molecule’s conformational flexibility allows steric complementarity with binding sites. Compared to other isomers (e.g., 1,4- or 4,5-DCQA), the 3,4 configuration balances steric strain and hydrophilicity, maximizing interaction diversity through both hydrophobic (aromatic) and hydrophilic (hydroxyl, ester) regions. This combination of functional groups and spatial arrangement enables broad interaction capacity, making dicaffeoylquinic acid IV highly effective in forming strong binding interactions in biological matrices. The number of hydrogen bond interactions ranged from 0 to 7, with compound Dicaffeoylquinic acid having the most at 7 interactions. The distances of these hydrogen bonds vary from 2.70 Å to 3.33 Å. Key interacting residues include Val70, Gly82, Gly92, Val94, Ala90, Glu91, Leu96, Arg86, Gly17, Arg95, and Lys22 (**Figure 5**). Previous studies have also demonstrated the potent antioxidant, anti-inflammatory, antimicrobial, and neuroprotective activities of Conyza and *Pseudo Conyza* extracts, which have been attributed to their rich phytochemical composition (Veber et al., 2002; Jung et al., 2009; Wong-Paz et al., 2015; Ouamnina et al., 2024). Furthermore, plant-derived metabolites like dicaffeoylquinic acid and flavonoids, widely targeted metabolite modificomics technologies have emerged as powerful tools for enhancing the analysis of complex biological samples. Technologies like targeted liquid chromatography-mass spectrometry (LC-MS) and stable isotope labeling empower researchers to concentrate on specific metabolite classes while expanding detection capabilities. This allows for the simultaneous identification and quantification of hundreds of compounds in a single assay. For example, modifications such as derivatization or enzymatic treatments enhance the ionization efficiency and stability of metabolites, facilitating their detection in diverse matrices like plant extracts. In these extracts, flavonoids, secondary metabolites recognized for their antioxidant and health-promoting properties are often found in low concentrations (Yang et al., 2024).

## 4 Conclusions

The present study demonstrates that *Pseudoconyza viscosa* (Mill.) is a significant source of phenolic and flavonoid compounds, as indicated by the significant concentrations of Quercetin-O-dihexoside and other bioactive metabolites identified through HPLC and UV-VIS spectroscopy. The total phenolic content (TPC) of 311.74 mg GAE/g and total flavonoid content (TFC) of 208.2 mg QE/g indicate the extract’s significant antioxidant capacity, aligning with its traditional use in medicinal applications. The presence of various aromatic compounds, including flavonoid glycosides and dicaffeoylquinic acids, suggests that *Pseudoconyza viscosa* (Mill.) exhibits a wide range of biological activities, including antioxidant, anti-inflammatory, and neuroprotective effects. The In-silico molecular docking studies further support these findings, demonstrating strong binding interactions between the identified phytochemicals and human peroxiredoxin 5, which may elucidate their mechanisms of action. In silico analysis against human peroxiredoxin 5 (PDB ID: 1HD2) revealed minimum binding energy of -7.8 kcal/mol for compounds Dicaffeoylquinic acid IV. The antioxidant activity of compounds Dicaffeoylquinic acid IV suggest the potential use of this compound as potential drug lead candidates which corroborate with the traditional uses of the *Pseudoconyza viscosa* (Mill.). Further research is required to fully comprehend the molecular processes underlying the effects of *Pseudoconyza viscosa (Mill.)* extract.

## Data Availability

The original contributions presented in the study are included in the article/supplementary material, further inquiries can be directed to the corresponding author/s.
